# The risk of incident atrial fibrillation in patients with type 2 diabetes treated with sodium glucose cotransporter-2 inhibitors, glucagon-like peptide-1 receptor agonists, and dipeptidyl peptidase-4 inhibitors: a nationwide cohort study

**DOI:** 10.1186/s12933-022-01549-x

**Published:** 2022-06-28

**Authors:** Yi-Hsin Chan, Tze-Fan Chao, Shao-Wei Chen, Hsin-Fu Lee, Pei-Ru Li, Wei-Min Chen, Yung-Hsin Yeh, Chi-Tai Kuo, Lai-Chu See, Gregory Y. H. Lip

**Affiliations:** 1 The Cardiovascular Department, Chang Gung Memorial Hospital, Linkou, Taoyuan, 33305 Taiwan; 2 College of Medicine, Chang Gung University, Taoyuan City, 33302 Taiwan; 3 School of Traditional Chinese Medicine, College of Medicine, Chang-Gung University, Taoyuan City, 33302 Taiwan; 4 Microscopy Core Laboratory, Chang Gung Memorial Hospital, Linkou, Taoyuan City, 33305 Taiwan; 5 Division of Cardiology, Department of Medicine, Taipei Veterans General Hospital, Taipei, Taiwan; 6 Institute of Clinical Medicine, Cardiovascular Research Center, National Yang Ming Chiao Tung University, Taipei, Taiwan; 7 Division of Thoracic and Cardiovascular Surgery, Department of Surgery, Chang Gung Memorial Hospital, Linkou Medical Center, Taoyuan City, Taiwan; 8 Graduate Institute of Clinical Medical Sciences, College of Medicine, Chang Gung University, Taoyuan City, Taiwan; 9 New Taipei City Municipal Tucheng Hospital (Chang Gung Memorial Hospital, Tucheng branch, New Taipei City, Taiwan; 10 Department of Public Health, College of Medicine, Chang Gung University, Taoyuan City, 33302 Taiwan; 11 Biostatistics Core Laboratory, Molecular Medicine Research Center, Chang Gung University, Taoyuan City, 33302 Taiwan; 12 Division of Rheumatology, Allergy and Immunology, Department of Internal Medicine, Chang Gung Memorial Hospital, Linkou, Taoyuan City, 33305 Taiwan; 13 Liverpool Centre for Cardiovascular Science, University of Liverpool and Liverpool Heart & Chest Hospital, Liverpool, UK; 14 Department of Clinical Medicine, Aalborg University, Aalborg, Denmark

**Keywords:** Atrial fibrillation, Type 2 diabetes mellitus, Sodium-glucose cotransporter-2 inhibitor, Glucagon-like peptide-1 receptor agonist, Dipeptidyl peptidase-4 inhibitor

## Abstract

**Background:**

Although a few meta-analyses were conducted to compare the risk of incident atrial fibrillation (AF) between sodium-glucose cotransporter-2 inhibitor (SGLT2i), glucagon-like peptide-1 receptor agonists (GLP-1RA), and other anti-hyperglycemic agents using indirect or direct comparison, the above analyses showed conflicting results with each other. We aimed to evaluate the risk of new-onset AF associated with the use of SGLT2i, GLP-1RA, and dipeptidyl peptidase-4 inhibitor (DPP4i) among a large longitudinal cohort of diabetic patients.

**Methods:**

In this nationwide retrospective cohort study based on the Taiwan National Health Insurance Research Database, a total of 344,893, 44,370, and 393,100 consecutive patients with type 2 diabetes without preexisting AF receiving GLP-1RA, SGLT2i, and DPP4i, respectively, were enrolled from May 1, 2016, to December 31, 2019. We used 1:1 propensity score matching (PSM) to balance covariates across paired study groups. Patients were followed from the drug index date until the occurrence of AF, death, discontinuation of the index drug, or the end of the study period (December 31, 2020), whichever occurred first.

**Results:**

After PSM, there were 245,442, 43,682, and 39,190 paired cohorts of SGLT2i-DPP4i, SGLT2i-GLP-1RA, and GLP-1RA-DPP4i, respectively. SGLT2i treatment was associated with lower risk of new-onset AF in participants with type 2 diabetes compared with either DPP4i [hazard ratio (HR):0.90; 95% confidential interval (CI) 0.84–0.96; *P* = 0.0028] or GLP-1RA [HR 0.74; 95% CI 0.63–0.88; *P* = 0.0007] treatment after PSM. There was no difference in the risk of incident AF between GLP-1RA and DPP4i users [HR 1.01; 95% CI 0.86–1.19; *P* = 0.8980]. The above findings persisted among several important subgroups. Dapagliflozin was specifically associated with a lower risk of new-onset AF compared with DPP4i (*P* interaction = 0.02).

**Conclusions:**

Compared with DPP4i, SGLT2i but not GLP-1RA was associated with a lower risk of incident AF in patients with type 2 diabetes.

**Supplementary Information:**

The online version contains supplementary material available at 10.1186/s12933-022-01549-x.

## Background

Type 2 diabetes mellitus and atrial fibrillation (AF) represent common chronic clinical disease burdens and the ever-aging population globally [1]. Furthermore, diabetes is closely related to AF, and their coexistence is associated with substantial risks of adverse cardiovascular events, hospitalization for heart failure, morbidity, and mortality [1–4]. Diabetes itself also has a causal relationship with AF, being an independent risk factor for the development of incident AF [5, 6]. The underlying pathogenic mechanisms of the development of AF in diabetes include electrical-electromechanical, structural and autonomic remodeling, forming the substrate for AF development and maintenance [1]. Insulin resistance or obesity, even without overt diabetes (e.g., glycated hemoglobin levels < 6.5%), is associated with a proinflammatory state, atrial dilatation, pericardial fat accumulation, and autonomic dysfunction are all known to increase the risk of AF [1, 6, 7]. Furthermore, ischemic cerebrovascular and cardiovascular disease and heart failure (HF) can increase the risk for the development of AF in several ways [8, 9]. Therefore, glucose-lowering agents that lead to loss of body weight, improvement of insulin resistance, and cardiovascular outcomes may be associated with decreased risk of incident AF. Clinical studies have reported that both sodium-glucose cotransporter-2 inhibitors (SGLT2i) and glucagon-like peptide-1 receptor agonists (GLP-1RA) reduce body weight and risks of major adverse cardiovascular events (MACE) and/or hospitalization for HF in patients with type 2 diabetes [10–18]. On the other hand, dipeptidyl peptidase-4 inhibitors (DPP4i) are generally safe and well-tolerated glucose-lowering agents that have been associated with a significant reduction in blood pressure and do not increase body weight [18–21]. Although several animal and pre-clinical studies demonstrated a cardioprotective effect of DPP4i [22, 23], these have not been clearly translated into clinically significant results in landmark cardiovascular outcome trials [19]. Also, whether these drugs are associated with a lower risk of AF remains unclear. Recent meta-analyses comparing the risk of incident atrial fibrillation (AF) between users of SGLT2i, GLP1-RA, and placebo (DPP4i and other anti-hypoglycemic agents) have shown conflicting results [24–29]. Some analyses have indicated that DPP4i treatment was associated with a higher risk for atrial arrhythmias [29, 30]. To date, a direct comparison between SGLT2i, GLP-1RA, and DPP4i regarding the risk of incident AF has not been conducted. We, therefore, aimed to investigate the risk of new-onset incident AF between DPP4i, GLP-1RA, and SGLT2i users using direct head-to-head comparisons, specifically focused on the Asian population with type 2 diabetes, in a large real-world setting.

## Methods

### Database

We performed a nationwide retrospective cohort study using the Taiwan National Health Insurance Research Database (NHIRD), which contains detailed health-care information for more than 23 million enrollees with a > 99% coverage rate of residents of Taiwan [31]. Informed consent was waived in the present study because the original identification number of each patient in the NHIRD had been encrypted and de-identified to protect their privacy. The study was approved by the Institutional Review Board of the Chang Gung Medical Foundation (201801427B0, and 201802075B0). The interpretation and conclusions contained herein do not represent the position of Chang Gung Memorial Hospital or the National Health Insurance Administration in Taiwan.

### Study cohort

**Fig. 1:**
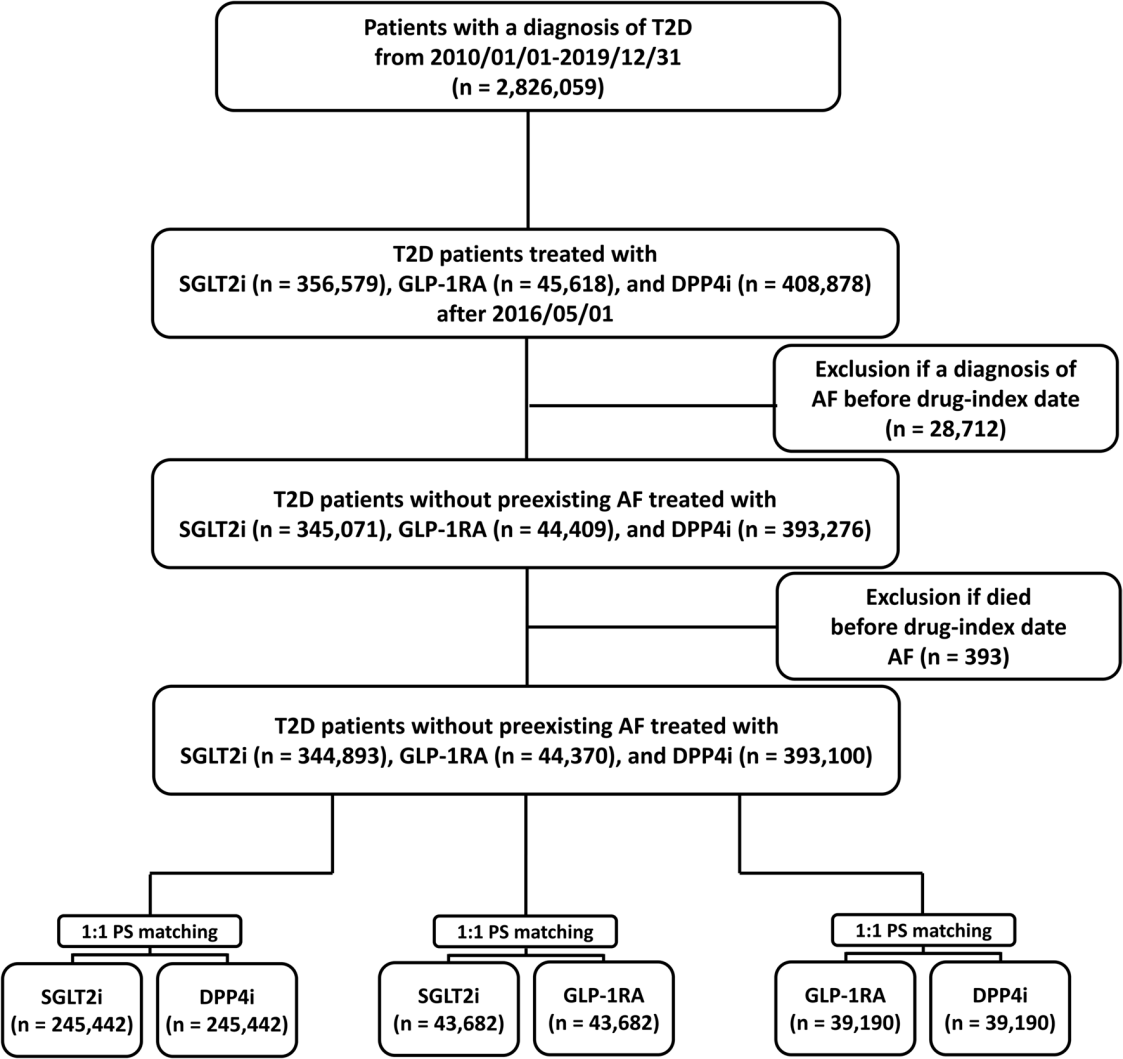
Enrollment of patients with type 2 diabetes (T2D) treated with sodium-glucose cotransporter 2 inhibitors (SGLT2i), glucagon-like peptide-1 receptor agonist (GLP-1RA), and dipeptidyl peptidase-4 inhibitors (DPP4i). A total of 344,893, 44,370, and 393,100 patients with T2D without pre-existing atrial fibrillation (AF) treated with SGLT2i, GLP-1RA, and DPP4i from May 1, 2016 to December 31, 2019, respectively, were enrolled in the present study. There were 245,442, 43,682, and 39,190 paired cohorts of SGLT2i versus DPP4i, SGLT2i versus GLP-1RA, and GLP-1RA versus DPP4i, respectively, after propensity score matching (PSM)

The study identified a total of 2,826,059 patients with type 2 diabetes diagnosed using the *International Classification of Diseases (ninth revision) Clinical Modification* (*ICD-10-CM codes* (E10.0, E10.1, E10.9, E11.0, E11.1, and E11.9) between January 1, 2010, and December 31, 2019. Because the SGLT2i was the latest drug approval (May 1, 2016) of GLP-1RA, SGLT2i, and DPP4i in Taiwan, the drug index date was defined as the first date of prescription for GLP-1RA, SGLT2i, or DPP4i after May 1, 2016, to achieve a head-to-head comparison at the same time. There were 356,579 and 45,618 patients receiving first prescriptions of SGLT2i (Empagliflozin and dapagliflozin; approved on May 1, 2016, in Taiwan. Canagliflozin; approved on March 1, 2018. Ertugliflozin; approved on July 1, 2019) and GLP1-RA (dulaglutide, exenatide, liraglutide, lixisenatide, or semaglutide) after May 1, 2016, respectively. Of the other 2,423,862 patients not receiving SGLT2i or GLP-1RA treatments, there were 408,878 patients receiving first prescriptions for DPP4i (alogliptin linagliptin, saxagliptin, sitagliptin, or vildagliptin,) during the same period. Patients with type 2 diabetics cannot use SGLT2i, GLP-1RA, and DPP4i simultaneously (or add-on) according to Taiwan’s NHI regulations for financial consideration. Patients with AF diagnosis before index-drug were excluded (n = 28,712). We also excluded those patients who died before the drug-index date (n = 393). Finally, 344,893, 44,370, and 393,100 diabetic patients without preexisting AF treated with SGLT2i, GLP1-RA, and DPP4i after May 1, 2016, enrolled in the present study. The flowchart of study enrollment is summarized in Fig. 1.

### Covariates

Baseline covariates were obtained from all claim records with diagnoses, procedures, or medication codes before the index date. A history of all prescription medications was confined to medications used at least once within 3 months before the index date. The ICD-10-CM codes used to identify baseline covariates are summarized in Additional file 1: Tables S1.

### Outcomes

The main study outcome was new-onset AF (ICD-10-CM code I48) diagnosed in accordance with one inpatient or at least two outpatient diagnostic codes assigned after the drug index date. Patients were followed from the drug-index date until the development of incident AF, discontinuation of index drug, mortality, or the end of the study period (December 31, 2020), whichever occurred first.

### Statistical analysis

The propensity score matching (PSM) method compares the risk of incident AF between the paired group of SGLT2i versus DPP4i, SGLT2i versus GLP-1RA, and GLP-1RA versus DPP4i, respectively. The PSM re-weights the untreated group so that matched sets of treated and untreated subjects are similar in demographic, comorbidity, and medication [32]. We calculated the propensity score, the predicted probability of treatment conditional on all the covariates in Table 1, using the generalized boosted model (GBM). The GBM involves an iterative process with multiple regression trees to capture complex and nonlinear relationships between treatment assignment and the pretreatment covariates without over-fitting the data and leading to the best balance between study groups [33]. The PSM ratio between the SGLT2i versus DPP4i, SGLT2i versus GLP-1RA, and GLP-1RA versus DPP4i was 1:1 without replacement and nearest neighbor matching within a caliper width (8-to-1 digit matching) [34]. The balance of potential confounders at the baseline (index date) between paired study groups was assessed using the absolute standardized mean difference (ASMD) rather than statistical testing because balance is a property of the sample and not of the underlying population. An ASMD value of ≤ 0.1 indicates an insignificant difference in potential confounders between the two paired study groups [35]. Incidence rates were estimated using the total number of study outcomes during the follow-up period divided by person-years at risk. The risk of study outcomes occurring over the follow-up duration for SGLT2i versus DPP4i, SGLT2i versus GLP-1RA, and GLP-1RA versus DPP4i was obtained using survival analysis. The cumulative incidence curve of AF between the paired study groups was plotted using the Kaplan–Meier method and compared with hazard ratios (HRs) and 95% CIs using the Cox proportional hazards model. Noted that only the paired study grouping was included in the Cox model because the paired study groups were well balanced in baseline characteristics after PSM [32]. The cause- specific hazard model which account for deaths as competing risk events was also made to estimate the subdistribution HR of new-onset AF between the paired study groups [36]. Subgroup analysis was made to determine whether the risk of AF between the three paired cohorts remained in specific subgroups. Statistical significance was defined as a *p* value of < 0.05. All statistical analyses were performed using SAS 9.4 (SAS Institute Inc., Cary, NC, USA).

**Table 1:** Clinical characteristics of the patients with type 2 diabetes treated with SGLT2i, GLP-1RA, and DPP4i before and after propensity score matching (PSM)

Baseline characteristics	Before PSM				After PSM			After PSM			After PSM		
	SGLT2i (n = 344,893)	GLP-1RA (n = 44,370)	DPP4i (n = 393,100)	Max ASMD	SGLT2i (n = 245,442)	DPP4i (n = 245,442)	ASMD	SGLT2i (n = 43,682)	GLP-1RA (n = 43,682)	ASMD	GLP-1RA (n = 39,190)	DPP4i (n = 39,190)	ASMD
Age (mean ± SD)	58.6 ± 12.1	55.3 ± 13.2	63.0 ± 13.2		59.0 (12.8)	60.3 (11.7)		56.8 (11.9)	55.2 (13.2)		55.4 (13.5)	58.6 (11.3)	
< 65 y/o	233,520 (67.71)	32,834 (74.00)	214,564 (54.58)	0.447	157,801 (64.29)	159,193 (64.86)	0.035	33,160 (75.91)	32,434 (74.25)	0.088	28,492 (72.70)	29,092 (74.23)	0.094
65–74 y/o	79,935 (23.18)	8175 (18.42)	99,054 (25.20)		59,845 (24.38)	59,794 (24.36)		7626 (17.46)	7984 (18.28)		7425 (18.95)	7197 (18.36)	
75–84 y/o	27,000 (7.83)	2901 (6.54)	58,700 (14.93)		23,522 (9.58)	22,579 (9.20)		2507 (5.74)	2817 (6.45)		2814 (7.18)	2509 (6.40)	
≥ 85 y/o	4438 (1.29)	460 (1.04)	20,782 (5.29)		4274 (1.74)	3876 (1.58)		389 (0.89)	447 (1.02)		459 (1.17)	392 (1.00)	
Male	201,889 (58.54)	22,834 (51.46)	214,352 (54.53)	0.143	138,611 (56.47)	138,350 (56.37)	0.002	22,333 (51.13)	22,517 (51.55)	0.008	20,440 (52.16)	20,355 (51.94)	0.004
Chronic lung disease	8215 (2.38)	1462 (3.30)	14,065 (3.58)	0.070	5749 (2.34)	5604 (2.28)	0.004	1243 (2.85)	1417 (3.24)	0.023	1256 (3.20)	1178 (3.01)	0.012
Chronic liver disease	61,198 (17.74)	8322 (18.76)	54,461 (13.85)	0.133	38,880 (15.84)	39,177 (15.96)	0.003	8179 (18.72)	8241 (18.87)	0.004	7041 (17.97)	6943 (17.72)	0.007
Chronic kidney disease	55,458 (16.08)	11,254 (25.36)	63,786 (16.23)	0.231	36,689 (14.95)	36,301 (14.79)	0.004	10,153 (23.24)	10,576 (24.21)	0.023	9157 (23.37)	8768 (22.37)	0.024
Congestive heart failure	3378 (0.98)	756 (1.70)	3439 (0.87)	0.074	1629 (0.66)	1666 (0.68)	0.002	575 (1.32)	657 (1.50)	0.016	568 (1.45)	541 (1.38)	0.006
Hypertension	240,837 (69.83)	32,220 (72.62)	245,978 (62.57)	0.216	163,972 (66.81)	163,490 (66.61)	0.004	31,190 (71.40)	31,549 (72.22)	0.018	27,854 (71.07)	27,634 (70.51)	0.012
Dyslipidemia	290,201 (84.14)	38,868 (87.60)	275,391 (70.06)	0.440	197,217 (80.35)	197,488 (80.46)	0.003	38,283 (87.64)	38,219 (87.49)	0.004	33,741 (86.10)	34,001 (86.76)	0.019
Diabetic ulcer	2258 (0.65)	614 (1.38)	2806 (0.71)	0.073	1469 (0.60)	1436 (0.59)	0.002	501 (1.15)	547 (1.25)	0.010	467 (1.19)	435 (1.11)	0.008
Stroke	12,758 (3.70)	1721 (3.88)	21,150 (5.38)	0.081	9637 (3.93)	9431 (3.84)	0.004	1531 (3.50)	1676 (3.84)	0.018	1577 (4.02)	1495 (3.81)	0.011
Ischemic heart disease	23,570 (6.83)	2936 (6.62)	13,116 (3.34)	0.160	9808 (4.00)	10,232 (4.17)	0.009	2557 (5.85)	2809 (6.43)	0.024	2226 (5.68)	2119 (5.41)	0.012
Chronic gout	52,487 (15.22)	7242 (16.32)	59,616 (15.17)	0.032	37,704 (15.36)	37,865 (15.43)	0.002	6748 (15.45)	7002 (16.03)	0.016	6430 (16.41)	6270 (16.00)	0.011
Peripheral artery disease	704 (0.20)	209 (0.47)	863 (0.22)	0.046	422 (0.17)	411 (0.17)	0.001	155 (0.35)	186 (0.43)	0.011	169 (0.43)	163 (0.42)	0.002
Malignancy	19,538 (5.66)	2430 (5.48)	32,100 (8.17)	0.107	15,226 (6.20)	14,840 (6.05)	0.007	2219 (5.08)	2390 (5.47)	0.018	2234 (5.70)	2127 (5.43)	0.012
History of bleeding	1264 (0.37)	230 (0.52)	3784 (0.96)	0.073	1079 (0.44)	987 (0.40)	0.006	191 (0.44)	219 (0.50)	0.009	218 (0.56)	214 (0.55)	0.001
History of PCI	23,625 (6.85)	2895 (6.52)	12,230 (3.11)	0.173	9151 (3.73)	9612 (3.92)	0.010	2461 (5.63)	2759 (6.32)	0.029	2180 (5.56)	2084 (5.32)	0.011
History of CABG	1984 (0.58)	337 (0.76)	1238 (0.31)	0.061	840 (0.34)	866 (0.35)	0.002	283 (0.65)	315 (0.72)	0.009	249 (0.64)	243 (0.62)	0.002
APT	96,424 (27.96)	12,021 (27.09)	92,230 (23.46)	0.103	58,905 (24.00)	59,184 (24.11)	0.003	11,063 (25.33)	11,696 (26.78)	0.033	10,334 (26.37)	9927 (25.33)	0.024
ACEI/ARB	198,252 (57.48)	25,822 (58.20)	193,019 (49.10)	0.183	132,347 (53.92)	132,130 (53.83)	0.002	25,031 (57.30)	25,348 (58.03)	0.015	22,289 (56.87)	22,120 (56.44)	0.009
ARNI	1590 (0.46)	185 (0.42)	465 (0.12)	0.064	367 (0.15)	410 (0.17)	0.004	150 (0.34)	179 (0.41)	0.011	123 (0.31)	122 (0.31)	0.001
Beta blocker	103,432 (29.99)	13,027 (29.36)	101,697 (25.87)	0.092	66,552 (27.12)	66,650 (27.16)	0.001	12,040 (27.56)	12,639 (28.93)	0.031	11,203 (28.59)	10,849 (27.68)	0.020
Loop diuretics	17,484 (5.07)	3472 (7.83)	30,088 (7.65)	0.112	12,256 (4.99)	11,938 (4.86)	0.006	2817 (6.45)	3164 (7.24)	0.032	3095 (7.90)	2874 (7.33)	0.021
MRA	11,098 (3.22)	1525 (3.44)	13,938 (3.55)	0.018	6608 (2.69)	6491 (2.64)	0.003	1292 (2.96)	1480 (3.39)	0.025	1337 (3.41)	1243 (3.17)	0.013
Nitrate	16,002 (4.64)	2180 (4.91)	17,124 (4.36)	0.027	9568 (3.90)	9635 (3.93)	0.001	1852 (4.24)	2060 (4.72)	0.023	1872 (4.78)	1789 (4.56)	0.010
Non-DHP CCB	10,068 (2.92)	1245 (2.81)	12,216 (3.11)	0.018	6462 (2.63)	6412 (2.61)	0.001	1096 (2.51)	1219 (2.79)	0.018	1113 (2.84)	1017 (2.60)	0.015
Digoxin	1986 (0.58)	205 (0.46)	2604 (0.66)	0.027	1239 (0.50)	1197 (0.49)	0.002	174 (0.40)	203 (0.46)	0.010	182 (0.46)	172 (0.44)	0.004
DHP CCB	145,011 (42.05)	18,226 (41.08)	169,847 (43.21)	0.043	100,712 (41.03)	107,373 (43.75)	0.055	18,132 (41.51)	17,796 (40.74)	0.016	15,802 (40.32)	17,991 (45.91)	0.113
Statin	235,174 (68.19)	32,023 (72.17)	207,551 (52.80)	0.408	152,641 (62.19)	152,713 (62.22)	0.001	31,443 (71.98)	31,469 (72.04)	0.001	27,162 (69.31)	27,087 (69.12)	0.004
NSAIDs	79,453 (23.04)	10,719 (24.16)	100,346 (25.53)	0.058	59,192 (24.12)	59,463 (24.23)	0.003	10,650 (24.38)	10,572 (24.20)	0.004	9775 (24.94)	9768 (24.92)	0.000
PPI	19,067 (5.53)	2669 (6.02)	31,754 (8.08)	0.101	14,558 (5.93)	14,277 (5.82)	0.005	2336 (5.35)	2603 (5.96)	0.027	2553 (6.51)	2313 (5.90)	0.025
H2 blocker	63,552 (18.43)	8123 (18.31)	87,960 (22.38)	0.101	46,910 (19.11)	46,947 (19.13)	< .001	7539 (17.26)	7931 (18.16)	0.024	7494 (19.12)	7299 (18.62)	0.013
Anti-diabetic agent													
Metformin	198,656 (57.60)	25,551 (57.59)	245,340 (62.41)	0.099	157,015 (63.97)	158,769 (64.69)	0.015	25,842 (59.16)	25,513 (58.41)	0.015	23,305 (59.47)	23,736 (60.57)	0.023
Sulfonylurea	203,927 (59.13)	26,406 (59.51)	170,955 (43.49)	0.325	131,131 (53.43)	130,340 (53.10)	0.007	26,423 (60.49)	26,227 (60.04)	0.009	23,314 (59.49)	23,593 (60.20)	0.015
Glinide	15,568 (4.51)	4300 (9.69)	24,902 (6.33)	0.203	11,696 (4.77)	11,478 (4.68)	0.004	3648 (8.35)	3806 (8.71)	0.013	3634 (9.27)	3623 (9.24)	0.001
Acarbose	41,010 (11.89)	7037 (15.86)	28,343 (7.21)	0.273	21,909 (8.93)	22,173 (9.03)	0.004	6788 (15.54)	6888 (15.77)	0.006	5879 (15.00)	5959 (15.21)	0.006
Glitazone	63,680 (18.46)	10,281 (23.17)	30,652 (7.80)	0.435	27,968 (11.39)	28,016 (11.41)	0.001	10,092 (23.10)	10,067 (23.05)	0.001	8178 (20.87)	8336 (21.27)	0.010
Insulin	58,063 (16.84)	23,232 (52.36)	70,838 (18.02)	0.805	35,077 (14.29)	34,991 (14.26)	0.001	22,653 (51.86)	22,553 (51.63)	0.005	18,147 (46.31)	18,048 (46.05)	0.005

Data are expressed as mean ± standard deviation (SD) or as percentage %

*ACEI*: angiotensin-converting enzyme inhibitor, *APT* anti-platelet agent, *ARB* angiotensin receptor blocker, *ARNI* angiotensin receptor-neprilysin inhibitor, *CABG* coronary artery bypass graft, *ASMD absolute* standardized mean difference, *CCB* calcium channel blocker, *DHP* dihydropyridine, *DPP4i* dipeptidyl peptidase-4 inhibitor, *GLP-1RA* glucagon-like peptide 1 receptor agonist, *MRA* mineralocorticoid receptor antagonist, *NSAID* non-steroidal anti-inflammatory drug, *PCI* percutaneous coronary intervention, *PPI* proton pump inhibitor, *PSM* propensity score matching, *SGLT2i* sodium glucose co-transporter-2 inhibitor

### Sensitivity analysis

We performed several sensitivity analyses to examine the robustness of the present results. First, some severe diabetic patients die before AF can occur. Therefore, the HR for risk of new-onset AF between the two paired study groups was analyzed after PSM and using death as a competing risk factor. Second, we repeated the analyses using new-onset AF with a hospital discharge diagnosis. Third, we repeated the analyses using the restricted AF outcome defined as the specified AF outcome with the consequent use of oral anticoagulant (OAC) and rhythm control management either using anti-arrhythmic drug, cardioversion, or catheter ablation after the AF outcome was established. Fourth, we considered the study groups with no previous SGLT2i, DPP4i, or GLP1-RA exposure based on a 1-year washout period. Fifth, SGLT2i use is contraindicated among individuals with end-stage kidney disease (ESKD). In sensitivity analysis, we excluded patients with ESKD (confirmed by both relevant ICD-9-CM or ICD-10-CM codes and enrollment in the Registry of Catastrophic Illness Patient Database, a subpart of the NHI database) to avoid confounding since ESKD is associated with an increased risk of AF. Indeed, insulin use may also be associated with an increased risk of AF [3, 37, 38]. Therefore, we excluded the study patients with concomitant use of insulin.

## Results

A total of 344,893, 44,370, and 393,100 patients treated with SGLT2i, GLP-1RA, and DPP4i were eligible for the present study. Among the SGLT2i users, 144,058 (41.8%), 179,004 (51.9%), 21,762 (6.3%), and 69 (0.0%) were treated with empagliflozin, dapagliflozin, canagliflozin, ertugliflozin, respectively. Among the GLP-1RA users, 20,677 (46.6%), 19,895 (44.9%), 3,432 (7.7%), 91 (0.2%), 275 (0.6%) were treated with liraglutide, dulaglutide, lixisenatide, exenatide, and semaglutide, respectively. Most of the DPP4i users were prescribed with sitagliptin (n = 125,767, 32.0%), followed by linagliptin (n = 119,537, 30.4%), vildagliptin (n = 114,713, 29.2%), saxagliptin (n = 28,625, 7.3%) and alogliptin (n = 4,458, 1.1%).

Table 1 summarizes the baseline demographic characteristics of the three groups before and after paired PSM. Before PSM, there were many differences in the baseline characteristics across the three study groups (most ASMD > 0.10). After PSM, there were 245,442, 43,682, and 39,190 paired cohorts of SGLT2i versus DPP4i, SGLT2i versus GLP-1RA, and GLP-1RA versus DPP4i, respectively: the three paired cohorts were well-balanced in most characteristics (ASMD < 0.10).

### AF incidence rates among the three study groups

Before PSM, there were 1964 (0.29 per 100 patient-years), 314 (0.32 per 100 patient-years), 3883 (0.46 per 100 patient-years) new-onset AF for the SGLT2i, GLP-1RA, DPP4i groups, respectively. DPP4i treatment were associated with a higher risk of new-onset AF in participants with type 2 diabetes compared with SGLT2i [HR 1.59; 95% confidential interval (CI) 1.51–1.68; *P* < 0.0001] and GLP-1RA [HR 1.44; 95% CI 1.28–1.61; *P* < 0.0001] before PSM, respectively.

### SGLT2i versus DPP4i after PSM

**Fig. 2:**
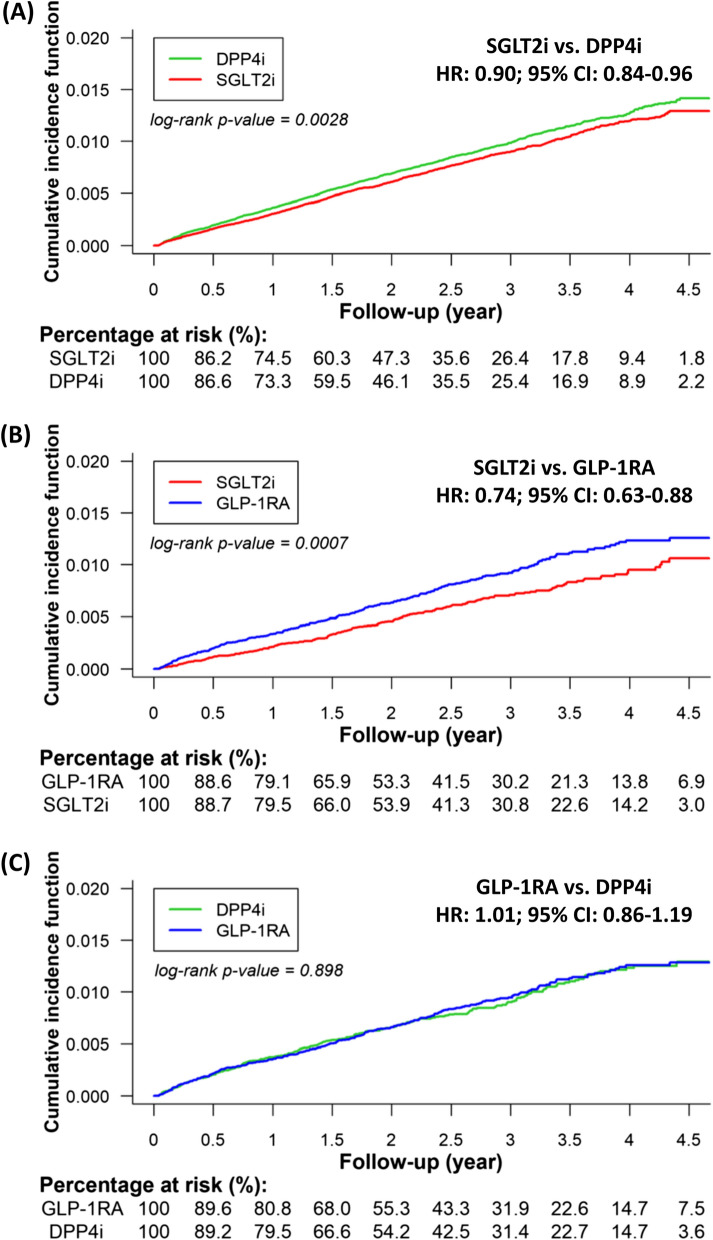
Cumulative risk of incident AF for the paired study cohorts treated with SGTL2i versus DPP4i (**A)**, SGLT2i versus GLP-1RA (**B**), and GLP-1RA versus DPP4i (**C**) after PSM. There was a clear separation of event curves for new-onset AF between the paired SGLT2i and DPP4i group and the paired SGLT2i and GLP-1RA group after PSM adjustment. SGLT2i treatment was associated with a lower risk of new-onset AF in participants with type 2 diabetes compared with DPP4i or GLP-1RA treatment after PSM. Conversely, there was no difference in the risk of incident AF between the GLP-1RA and DPP4i treatment

After PSM, For the 245,442 paired cohort of SGLT2i and DPP4i, the mean follow-up periods for the paired SGLT2i and DPP4i groups were 2.03 $\pm$ 1.29 and 2.01 $\pm$ 1.28 years, respectively. During the follow-up, there were 1,518 (0.29 per 100 person-year) and 1671 (0.34 per 100 person-year) new-onset AF events in the SGLT2i and DPP4i groups, respectively period. There was a clear separation of event curves for new-onset AF between the paired SGLT2i and DPP4i group after PSM adjustment. After PSM, SGLT2i treatment was associated with lower risk of new-onset AF in participants with type 2 diabetes compared with DPP4i [HR 0.90; 95% CI 0.84–0.96; *P* = 0.0028] treatment (Fig. 2A).

### SGLT2i versus GLP-1RA after PSM

For the 43,682 paired cohort of SGLT2i and GLP-1RA, the mean follow-up periods for the SGLT2i and GLP-1RA groups were 2.24 $\pm$ 1.32 and 2.24 $\pm$ 1.34 years, respectively. There were 228 (0.23 per 100 person-year) and 305 (0.31 per 100 person-year) new-onset AF events in the SGLT2i and GLP-1RA groups, respectively. After PSM, SGLT2i treatment was associated with lower risk of new-onset AF in participants with type 2 diabetes compared with GLP-1RA [HR 0.74; 95% CI 0.63–0.88; *P* = 0.0007] (Fig. 2B).

### GLP-1RA versus DPP4i after PSM

For the 39,190 paired cohort of GLP-1RA and DPP4i, the mean following-up periods for the GLP-1RA and DPP4i groups were 2.30 $\pm$ 1.34 and 2.26 $\pm$ 1.33 years, respectively. There were 289 (0.32 per 100 person-year) and 283 (0.32 per 100 person-year) new-onset AF events in the SGLT2i and GLP-1RA groups, respectively. There was no difference in the risk of incident AF between the GLP-1RA and DPP4i users [HR 1.01; 95% CI 0.86–1.19; *P* = 0.8980] (Fig. 2C).

### Sensitivity analyses

**Fig. 3:**
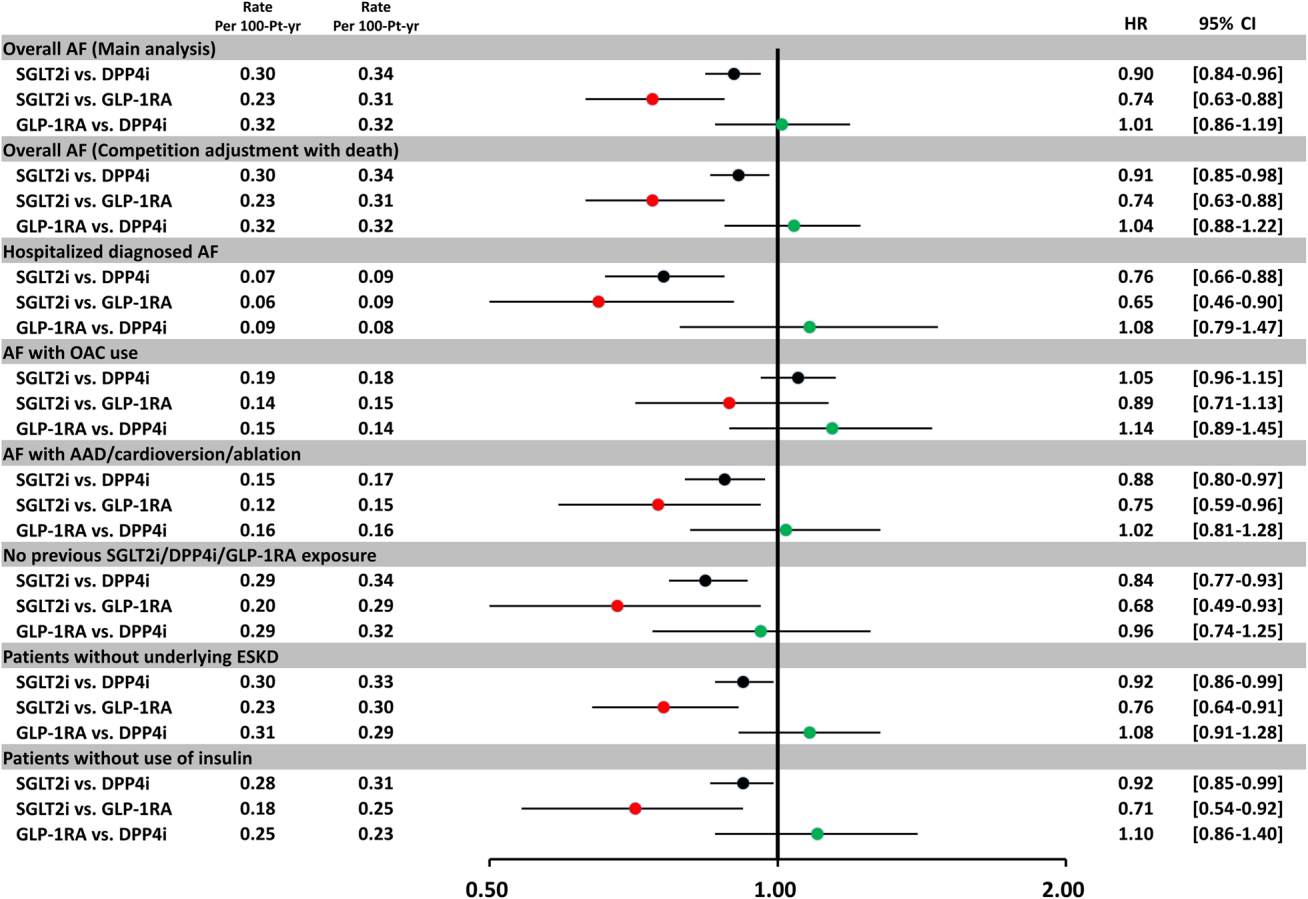
Sensitivity analyses of the hazard ratio for incident AF for the paired study cohorts treated with SGTL2i versus DPP4i, SGLT2i versus GLP-1RA, and GLP-1RA versus DPP4i after PSM. The sensitivity analyses showed the results were robust, and consistent with the main analysis. The use of SGLT2i was still associated with a lower risk of new-onset AF compared with either DPP4i or GLP-1RA after PSM, using death as a competing risk factor consistent with the main analysis. Specifically, we identified incident AF using either hospitalized diagnosed AF; AF with treatment using anti-arrhythmic drugs, cardioversion, or catheter ablation; or only patients without previous drug exposure, underlying ESKD, or concomitant use of insulin, the results remained consistent with the main analyses. There were no differences in the risk of incident AF with consequent use of oral anticoagulant treatment for three paired study groups

The use of SGLT2i was still associated with a lower risk of new-onset AF compared with either DPP4i or GLP-1RA after PSM, using death as a competing risk factor consistent with the main analyses. Specifically, when we identified incident AF using either hospitalized diagnosed AF; AF with treatment using anti-arrhythmic drugs, cardioversion, or catheter ablation; or only patients without previous drug exposure, underlying ESKD, or concomitant use of insulin, the results remained consistent with the main analyses. It is noted that there was no difference in the risk of incident AF with consequent use of OACs treatment for each paired study group (Fig. 3).

### Subgroup analysis

Subgroup analysis revealed that SGLT2i was associated with a lower risk of new-onset AF than DPP4i or GLP-1RA across most subgroups (Additional file 2: Figure S1, Additional file 3: Figure S2). Dapagliflozin was specifically associated with a lower risk of new-onset AF than DPP4i (*P* interaction = 0.02) (Additional file 2: Figure S1). In addition, the use of SGLT2i was associated with greater reductions in new-onset AF events in subgroups, including those without concomitant use of sulfonylurea, when compared with GLP-1RA (*P* interaction < 0.01) (Additional file 3: Figure S2). There were no differences in the risk of incident AF between the GLP-1RA and DPP4i in all subgroups (*P* interaction all > 0.05) (Additional file 4: Figure S3).

## Discussion

To the best of our knowledge, this is the largest observational study to specifically evaluate the risk of new-onset AF focused on an Asian population with type 2 diabetes treated with SGLT2i, GLP-1RA, and DPP4i. The main finding of this study was that SGLT2i treatment was associated with a lower risk of new-onset AF in participants with type 2 diabetes compared with either GLP-1RA or DPP4i treatment. Conversely, there was no difference in the risk of incident AF between the DPP4i and GLP-1RA. Furthermore, the above findings persisted among several important subgroups and sensitivity analyses.

Diabetes mellitus is an independent risk factor of stroke development in AF patients and an independent prognostic predictor for the development of AF [3, 4]. Nonetheless, there is conflicting literature concerning the association between AF and anti-diabetic medications. Several medications, such as insulin and sulfonylureas, have been associated with an increased risk of AF, possibly through hypoglycemia and glycemic fluctuation, and stimulation of the sympathetic nervous system is associated with an increased incidence of AF [3, 37, 38]. By contrast, other medications such as metformin, pioglitazone, and DPP4i may reduce the degree of atrial remodeling as an upstream therapy to prevent the development of AF [39–42]. In contrast to the treatment benefit of AF reduction for pioglitazone and DPP4i observed in retrospective observational studies, no significant differences in the risk of new-onset AF with the use of pioglitazone were reported in the PROactive, RECORD, and BARI 2D trials [43–45]. Furthermore, the meta-analysis of landmark cardiovascular outcome trials (CVOTs) showed that DPP4i treatment did not significantly affect the risk for AF, while they were associated with a significant increase in the risk for atrial flutter (AFL) [30].

Several insulin-independent mechanisms include reducing blood pressure, body weight, uric acid, epicardial adipose tissue, interstitial volume, atrial dilatation, increasing serum magnesium, and promoting mitochondrial biogenesis, hinting that SGLT2i might reduce the incidence of AF development [46]. SGLT2i inhibits sodium–hydrogen exchange in cardiac myocytes, which has been linked to ameliorating myocardial hypertrophy, fibrosis, remodeling, and HF [47]. Preclinical data showed that treatment of SGLT2i inhibits sympathetic overdrive, which also plays an important role in the development and maintenance of AF [48, 49]. Post hoc analysis of DECLARE–TIMI 58 showed that dapagliflozin was significantly associated with a relative risk reduction of AF/AFL by 19% compared with placebo among participants with type 2 diabetes, which was consistent regardless of the established atherosclerotic cardiovascular disease, HF, or preexisting AF at baseline [50]. However, because atrial tachyarrhythmia was not a predefined and monitored outcome, the risk reduction in AF/AFL associated with dapagliflozin versus placebo has not been reported consistently for other SGLT2is, such as the case of empagliflozin and canagliflozin. Furthermore, the DAPA-HF trial indicated that dapagliflozin did not significantly reduce the risk of new-onset AF compared to patients with HF and reduced ejection fraction without AF at baseline [51]. Recently, a pooled meta-analysis of 32 trials reported that SGLT2i treatment was associated with a significant reduction in the risk of incident AF/AFL by 19% compared with the control (OR 0.81; 95% CI 0.69–0.95; *p* = 0.008) [27]. The average cumulative incidence of AF/AFL was around 3.6 per 1,000 patient-years. Further subgroup analysis showed that only dapagliflozin (10 trials) was associated with a significantly lower risk of AF/AFL (OR 0.74; 95% CI 0.60–0.91; *p* = 0.005). Conversely, empagliflozin (9 trials) was associated with no significant difference in risk (OR 1.17; 95% CI 0.75–1.82; *p* = 0.49), and canagliflozin (8 trials) was associated with a numerically but non-significant lower risk of AF/AFL (OR 0.81; 95% CI 0.60–1.08; *p* = 0.15) when compared with placebo [27]. The above findings are of particular interest as they are more in line with our present study showing that dapagliflozin was specifically associated with a lower risk of incident AF when compared with either DPP4i or GLP-1RA (Additional file 3: Figure S2, Additional file 4: Figure S3).

Several CVOTs have shown that human-based GLP-1RAs reduce MACE and mortality risks in patients with type 2 diabetes[13–17]. However, other cardiovascular outcomes such as the incidence of incident AF have not been thoroughly investigated in those CVOTs associated with GLP-1RAs. Currently, there is conflicting literature regarding the risk of AF associated with treating GLP-1RA. Potential underlying mechanisms of treatment benefit GLP1-RA include several nonglycemic effects, such as reductions in body weight and blood pressure; anti-fibrotic effects; and improvement of microcirculation, endothelial function, and conduction properties, all of which may reduce the development of AF [52]. Conversely, GLP-1RAs were associated with an elevated heart rate by around 2–8 beats per minute higher than that of the control group, which may be related to GLP-1RA leading to systemic vasodilation with subsequent reflex tachycardia or a direct effect of the GLP-1RA on the autonomic nervous system and/or sinus node [52–54]. Due to the important role in the initiation and maintenance of AF medicated by sympathetic overflow [55], this effect may raise the possibility of an increased risk of AF. Indeed, recent observational studies have shown that the use of GLP-1RA was independently associated with a higher risk of incident AF (HR 2.27; 95% CI 1.49–3.47) among participants with diabetes [29]. The post hoc analysis of the HARMONY trial also showed that albiglutide was associated with a greater risk of AF and tachyarrhythmia [56]. However, these results were not consistent across all the CVOTs regarding GLP-1RAs, and the pooled meta-analysis of CVOTs (the LEADER, SUSTAIN‐6, REWIND, HARMONY, ELIXA, and PIONEER trials) showed no significant differences in the risk of incident AF between GLP1‐RA and placebo among participants with type 2 diabetes (OR 0.93; 95% CI 0.70–1.23; *I*^2^ = 58%) [28]. Conversely, some meta-analyses indicated that GLP-1RA was associated with a lower risk of AF/AFL compared to other glucose-lowering agents in patients with type 2 diabetes [24, 26]. These above trials are limited by AF not being a predefined and monitored endpoint. Further prospective trials are warranted to confirm the potential arrhythmogenic or antiarrhythmic effect of GLP-1RAs and investigate further whether the effect is a drug-specific or class effect.

### Limitations

To date, direct comparisons of SGLT2i, GLP-1RA, and DPP4i regarding the risk of AF among patients with type 2 diabetes are scarce. Although a few network meta-analyses were aimed to compare the risk of incident AF between SGLT2i, GLP-1RA, and other anti-hypoglycemic agents, the above studies show conflicting results [24–29]. Also, the data from randomized and placebo-controlled studies may be incomplete because they were derived from the documentation of adverse effects and were not predefined for systematically identifying AF. Nevertheless, there are several limitations to our present study. First, this was a retrospective and observational study. The clinical characteristics of the patients were different across three study groups. Although we have adjusted for several important parameters relevant to clinical outcomes using PSM models, residual unmeasured confounders are probably present. Nevertheless, we suggest that future prospective randomized studies are needed to determine whether our findings apply to patients with type 2 diabetes treated with these drugs. Second, the NHIRD does not contain several important laboratory data such as body weight, glycohemoglobin (HbA1c), and serum creatinine, all of which may be associated with the risk of incident AF among participants with type 2 diabetes [57]. In addition, even with adjustment for CKD, the diagnosis of CKD by coding could not reflect the severity of renal disease, which may interfere with DPP4i, GLP-1RA, and SGLT2i selection for each patient. Third, the Taiwan National Health Insurance (NHI) Program only covers prescription of only one of DPP4i, GLP-1RA, or SGLT2i at the same time due to financial considerations (DPP4i, SGLT2i, and GLP1-RA are the relatively new and expensive anti-diabetic medications). If physicians prescribe two or three of these drugs simultaneously (or add-on) for patients, only one drug can be covered by the NHI program (and the drug will be therefore captured in the claims database), whereas the other one or two drugs should be paid by patients themselves (which will not be captured in the claims database). As obtaining the medical information of each participant outside the Taiwan NHIRD scheme is difficult, there would be a small number of patients in the specific group (e.g. SGLT2i, which was captured in the NHIRD) receiving other combination (or add-on) therapies outside the NHIRD (e.g., GLP1-RA and/or DPP4i, which was not captured in the NHIRD). Fourth, AF does not necessarily lead to hospitalization, nor is it always associated with extensive symptoms. AF can remain entirely asymptomatic. Therefore, detection bias due to common contacts with the healthcare system in one of the exposure groups could bias the results. Also, AF/AFL was not a predefined outcome lacking rigorous prospective collection and monitoring observed in the pivotal CVOT trials regarding the SGLT2i, GLP-1RA, or DPP4i. We call for further study regarding the clinical importance and reliability of this finding. Fifth, the present study was conducted based on an on-treatment design. It did not consider the changes in medical status or activity (e.g., new diagnosis of comorbidities or discontinuation/add-on of co-medication) during their follow-up period. Given that the mean follow-up was more than 2 years, time-dependent confounding could become critical. Finally, we only investigated Asian patients, and whether our result can be extrapolated to other ethnicities is unclear.

## Conclusions

SGLT2i treatment was associated with a lower risk of incident AF than DPP4i or GLP-1RA treatment among patients with type 2 diabetes, irrespective of underlying comorbidities in a large real-world setting. There was no difference in the risk of incident AF between the DPP4i and GLP-1RA treatment.

## Supplementary Information

**Additional file 1: Table S1 **International Classification of Diseases (10^th^ edition) Clinical Modification (ICD 10-CM) codes used to define comorbidities and clinical outcomes in this study.

**Additional file 2:  Figure S1. **Subgroup analysis of forest plot of hazard ratio (HR) for sodium-glucose cotransporter 2 inhibitors (SGLT2i) versus dipeptidyl peptidase-4 inhibitors (DPP4i) among patients with type 2 diabetes (T2D) after propensity score matching (PSM). Subgroup analysis revealed that use of SGLT2i was associated with a lower risk of new-onset AF compared with use of DPP4i across most subgroups. It is noted that dapagliflozin was specifically associated with a lower risk of new-onset AF compared with DPP4i (*P* interaction = 0.02).

**Additional file 3:  Figure S2. **Subgroup analysis of forest plot of HR for SGLT2i versus glucagon-like peptide-1 receptor agonist (GLP-1RA) among patients with T2D after PSM. Subgroup analysis revealed that use of SGLT2i was associated with a lower risk of new-onset AF compared with use of DPP4i across most subgroups. Use of SGLT2i was associated with greater reductions in new-onset AF events in subgroup including those without concomitant use of sulfonylurea when compared with GLP-1RA (*P* interaction < 0.01).

**Additional file 4:  Figure S3. **Subgroup analysis of forest plot of HR for GLP-1RA versus DPP4i among patients with T2D after PSM. There was no difference of the risk of incident AF between the GLP-1RA and DPP4i across all subgroups (*P* interaction > 0.05).

## Data Availability

We used the National Health Insurance Research Database Taiwan (NHIRD), which is only available in the Health and Welfare Data Science Center, Taiwan. We cannot make our research data accessible, discoverable, and usable.

## Abbreviations

AAD: Antiarrhythmic drug
ACEI: Angiotensin-converting enzyme inhibitor
AF: Atrial fibrillation
ARB: Angiotensin receptor blocker
ASMD: Absolute standardized mean difference
AMI: Acute myocardial infarction
APT: Antiplatelet agent
CKD: Chronic kidney disease
DM: Diabetes mellitus
DPP4i: Dipeptidyl peptidase-4 inhibitor
GLP-1RA: Glucagon-like peptide-1 receptor agonist
IHD: Ischemic heart disease
OAC: Oral anticoagulant
PAD: Peripheral artery disease
PSM: Propensity score matching
SGLT2i: Sodium glucose cotransporter-2 inhibitor
SU: Sulfonylurea
T2D: Type 2 diabetes
CI: Confidential interval
HR: Hazard ratio
PSM: Propensity score matching
ESKD: End stage kidney disease
