# Supplementary material for: The risk of incident atrial fibrillation in patients with type 2 diabetes treated with sodium glucose cotransporter-2 inhibitors, glucagon-like peptide-1 receptor agonists, and dipeptidyl peptidase-4 inhibitors: a nationwide cohort study
Source: Cardiovasc Diabetol. 2022 Jun 28;21:118. doi: 10.1186/s12933-022-01549-x (PMC9241240; doi:10.1186/s12933-022-01549-x)
Supplement: Supplementary file 1 — Additional file 1: Table S1 International Classification of Diseases (10th edition) Clinical Modification (ICD 10-CM) codes used to define comorbidities and clinical outcomes in this study. [file 12933_2022_1549_MOESM1_ESM.doc]

**ADDITIONAL FILE 1: MATERIAL**

**Additional Table S1**

***International Classification of Diseases (10^th^ edition) Clinical Modification* (ICD 10-CM) codes used to define comorbidities and clinical outcomes in this** study

| **Disease** | **ICD-10 Codes** | **Diagnosis definition** |
| --- | --- | --- |
| Ischemic stroke | I63, I64 | Discharge |
| Acute Myocardial infarction | I21-I23 | Discharge |
| Peripheral arterial disease | I70.0, I70.2, I70.9, I70.3, I70.8, I75.89, I70.9, I73.0, I73.1, I73.8, I73.9, I79.1, I79.8, I74.01, I74.09, I74.3, I74.4, I74.5, I74.8, I74.9, I77.9, I70.0, 41, 045, 047, 049, 04B, 04C, 04H, 04J, 04L, 04N, 04P, 04Q, 04R, 04S, 04U, 04V, 04W  Location:(C,D,E,F,H,J,K,L,M,N,P,Q,R,S,T,U,V,W,Y)  0Y67, 0Y68, 0Y6C, 0Y6D, 0Y6F, 0Y6G, 0Y6H, 0Y6J, 0Y6M, 0Y6N, 0Y6P, 0Y6Q, 0Y6R, 0Y6S, 0Y6T, 0Y6U, 0Y6V, 0Y6W, 0Y6X, 0Y6Y | Discharge or Outpatient department ≥2 |
| Ischemic heart disease | I21-I25 | Outpatient department ≥2 |
| Congestive heart failure | I11.0, I13.0, I13.2, I42.0, I50, I50.1, I50.9 | Discharge |
| Hypertension | I10-I16 | Outpatient department ≥2 |
| Diabetes mellitus | E11-E14 | Outpatient department ≥2 |
| Hyperlipidemia | E78 | Outpatient department ≥2 |
| Chronic gout | M10, M1A | Outpatient department ≥2 |
| Chronic lung disease | J41-J44 | Discharge |
| Chronic kidney disease | I12, I13, N00, N01, N02, N03, N04, N05, N07, N11, N14, N17, N18, N19, Q61 | Outpatient department ≥2 |
| Chronic liver disease | B150, B160, B162, B190, K704, K72, K766, I85 | Outpatient department ≥2 |
| Malignancy | C | Outpatient department ≥2 |
| Diabetic ulcer | E11.621 | Discharge or Outpatient department ≥2 |
| Diabetic ulcer:  Primary diagnosis of DM plus ulcer of lower limb | L97.901, L97.902, L97.903, L97.904, L97.909, L97.911, L97.912, L97.913, L97.914, L97.919, L97.921, L97.922, L97.923, L97.924, L97.929, L97.101, L97.102, L97.103, L97.104, L97.109, L97.111, L97.112, L97.113, L97.114, L97.119, L97.121, L97.122, L97.123, L97.124, L97.129, L97.201, L97.202, L97.203, L97.204, L97.209, L97.211, L97.212, L97.213, L97.214, L97.219, L97.221, L97.222, L97.223, L97.224, L97.229, L97.301, L97.302, L97.303, L97.304, L97.309, L97.311, L97.312, L97.313, L97.314, L97.319, L97.321, L97.322, L97.323, L97.324, L97.329, L97.401, L97.402, L97.403, L97.404, L97.409, L97.411, L97.412, L97.413, L97.414, L97.419, L97.421, L97.422, L97.423, L97.424, L97.429, L97.501, L97.502, L97.503, L97.504, L97.509, L97.511, L97.512, L97.513, L97.514, L97.519, L97.521, L97.522, L97.523, L97.524, L97.529, L97.801, L97.802, L97.803, L97.804, L97.809, L97.811, L97.812, L97.813, L97.814, L97.819 | Discharge or Outpatient department ≥2 |

**Additional Figure Legend**

**Additional Figure I**

**Subgroup analysis of forest plot of hazard ratio (HR) for sodium-glucose cotransporter 2 inhibitors (SGLT2i) versus dipeptidyl peptidase-4 inhibitors (DPP4i) among patients with type 2 diabetes (T2D) after propensity score matching (PSM).**

Subgroup analysis revealed that use of SGLT2i was associated with a lower risk of new-onset AF compared with use of DPP4i across most subgroups. It is noted that dapagliflozin was specifically associated with a lower risk of new-onset AF compared with DPP4i (*P* interaction = 0.02).

ACEI = angiotensin-converting enzyme inhibitor; APT = antiplatelet therapy; ARB = angiotensin receptor blocker; CI = confidential interval; CKD = chronic kidney disease; CVD = cardiovascular disease; DPP4 = dipeptidyl peptidase-4 inhibitor; HR = hazard ratio; PSM = propensity score matching; SGLT2i = sodium-glucose cotransporter 2 inhibitor; SU = sulfonyurea; T2D = type 2 diabetes; TZD = thiazolidinedione

Other abbreviations as in **Figure 1 and 2**

**Additional Figure II**

**Subgroup analysis of forest plot of HR for SGLT2i versus glucagon-like peptide-1 receptor agonist (GLP-1RA) among patients with T2D after PSM.**

Subgroup analysis revealed that use of SGLT2i was associated with a lower risk of new-onset AF compared with use of DPP4i across most subgroups. Use of SGLT2i was associated with greater reductions in new-onset AF events in subgroup including those without concomitant use of sulfonylurea when compared with GLP-1RA (*P* interaction < 0.01).

**GLP-1RA = glucagon-like peptide-1 receptor agonist**

**Other abbreviations as in Additional Figure I**

**Additional Figure III**

**Subgroup analysis of forest plot of HR for GLP-1RA versus DPP4i among patients with T2D after PSM.**

There was no difference of the risk of incident AF between the GLP-1RA and DPP4i across all subgroups (*P* interaction > 0.05).

**The abbreviations as in Additional Figure I and II**
