# Supplementary figures and images for: The risk of incident atrial fibrillation in patients with type 2 diabetes treated with sodium glucose cotransporter-2 inhibitors, glucagon-like peptide-1 receptor agonists, and dipeptidyl peptidase-4 inhibitors: a nationwide cohort study
Source: Cardiovasc Diabetol. 2022 Jun 28;21:118. doi: 10.1186/s12933-022-01549-x (PMC9241240; doi:10.1186/s12933-022-01549-x)

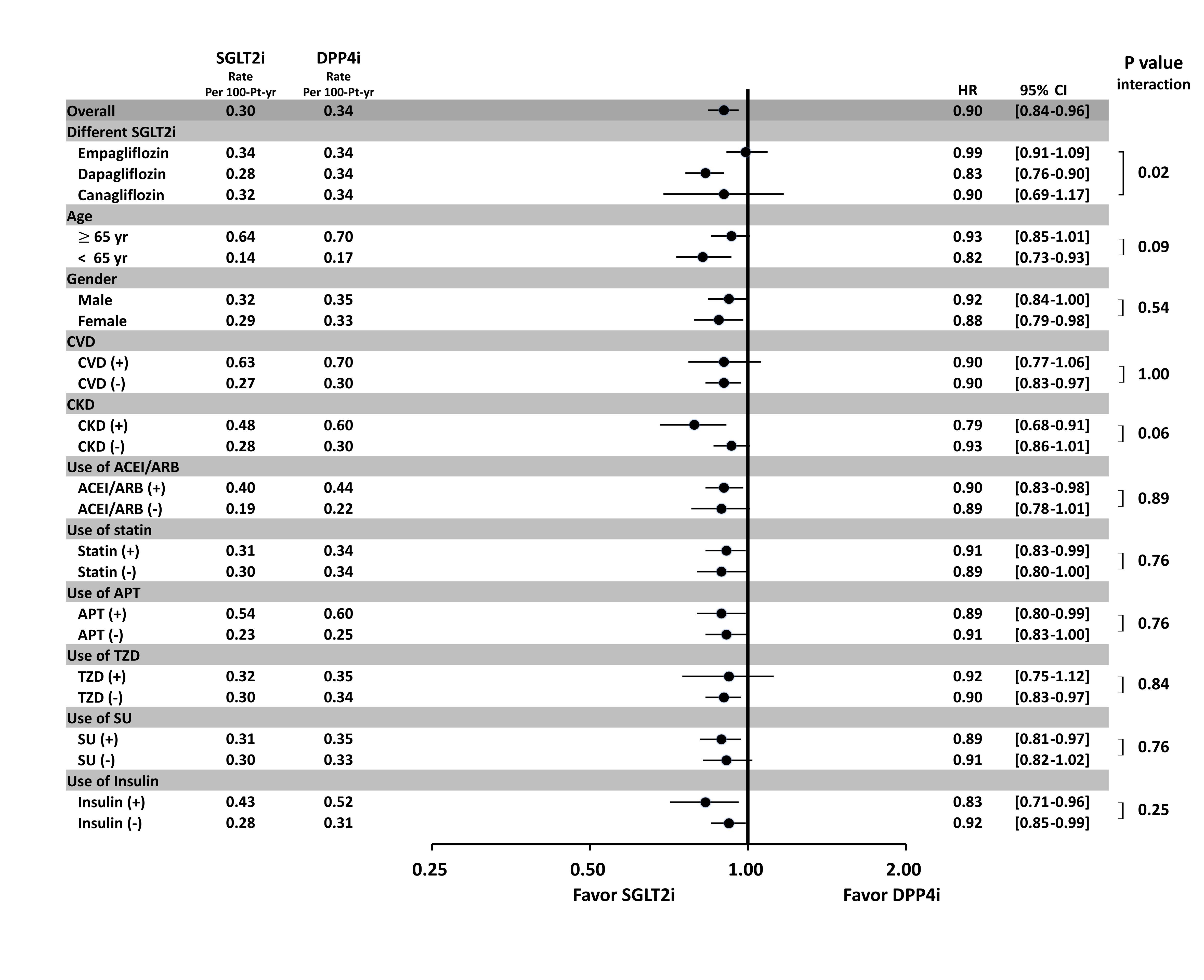

Supplement: Supplementary file 2 — Additional file 2: Figure S1. Subgroup analysis of forest plot of hazard ratio (HR) for sodium-glucose cotransporter 2 inhibitors (SGLT2i) versus dipeptidyl peptidase-4 inhibitors (DPP4i) among patients with type 2 diabetes (T2D) after propensity score matching (PSM). Subgroup analysis revealed that use of SGLT2i was associated with a lower risk of new-onset AF compared with use of DPP4i across most subgroups. It is noted that dapagliflozin was specifically associated with a lower risk of new-onset AF compared with DPP4i (P interaction = 0.02). [file 12933_2022_1549_MOESM2_ESM.tif]

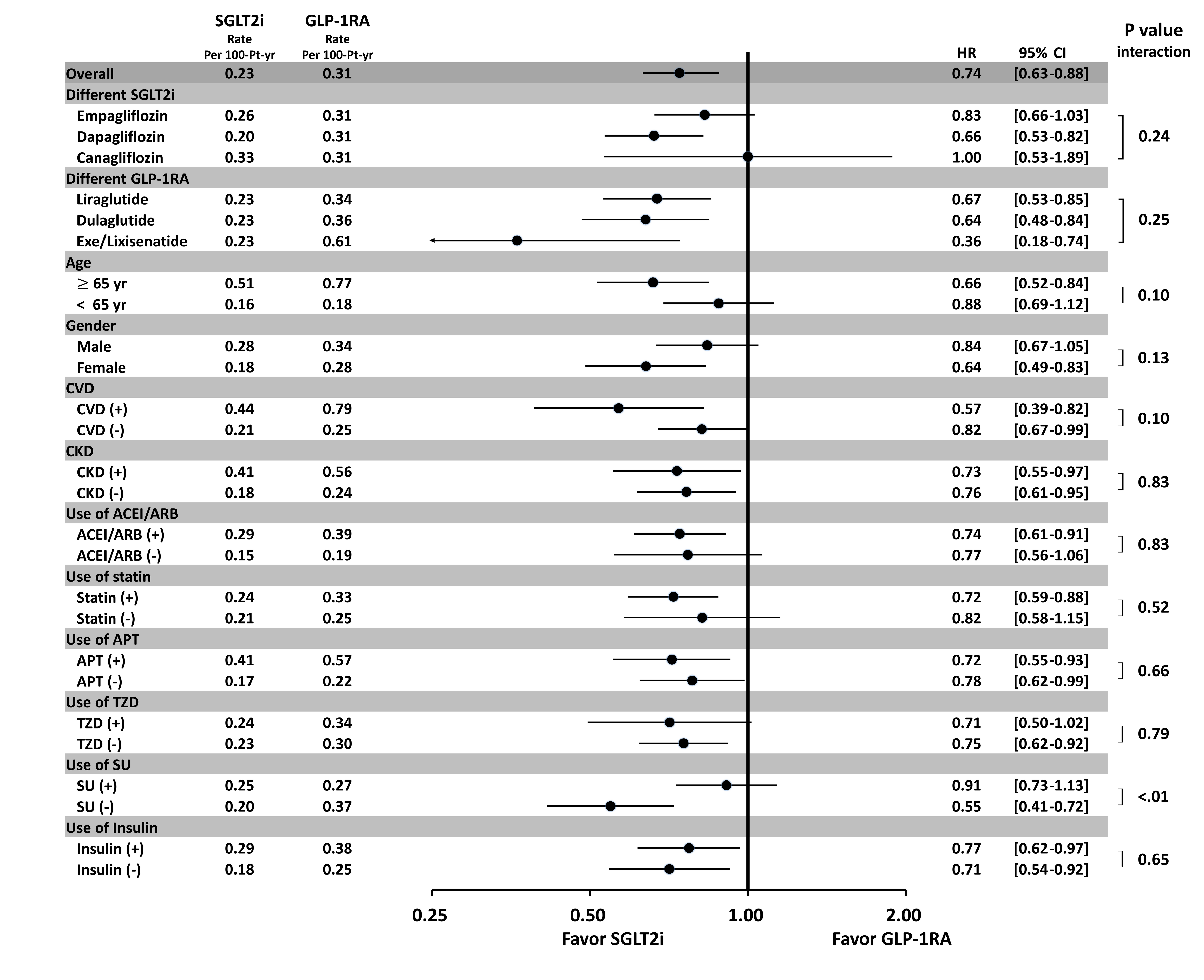

Supplement: Supplementary file 3 — Additional file 3: Figure S2. Subgroup analysis of forest plot of HR for SGLT2i versus glucagon-like peptide-1 receptor agonist (GLP-1RA) among patients with T2D after PSM. Subgroup analysis revealed that use of SGLT2i was associated with a lower risk of new-onset AF compared with use of DPP4i across most subgroups. Use of SGLT2i was associated with greater reductions in new-onset AF events in subgroup including those without concomitant use of sulfonylurea when compared with GLP-1RA (P interaction < 0.01). [file 12933_2022_1549_MOESM3_ESM.tif]

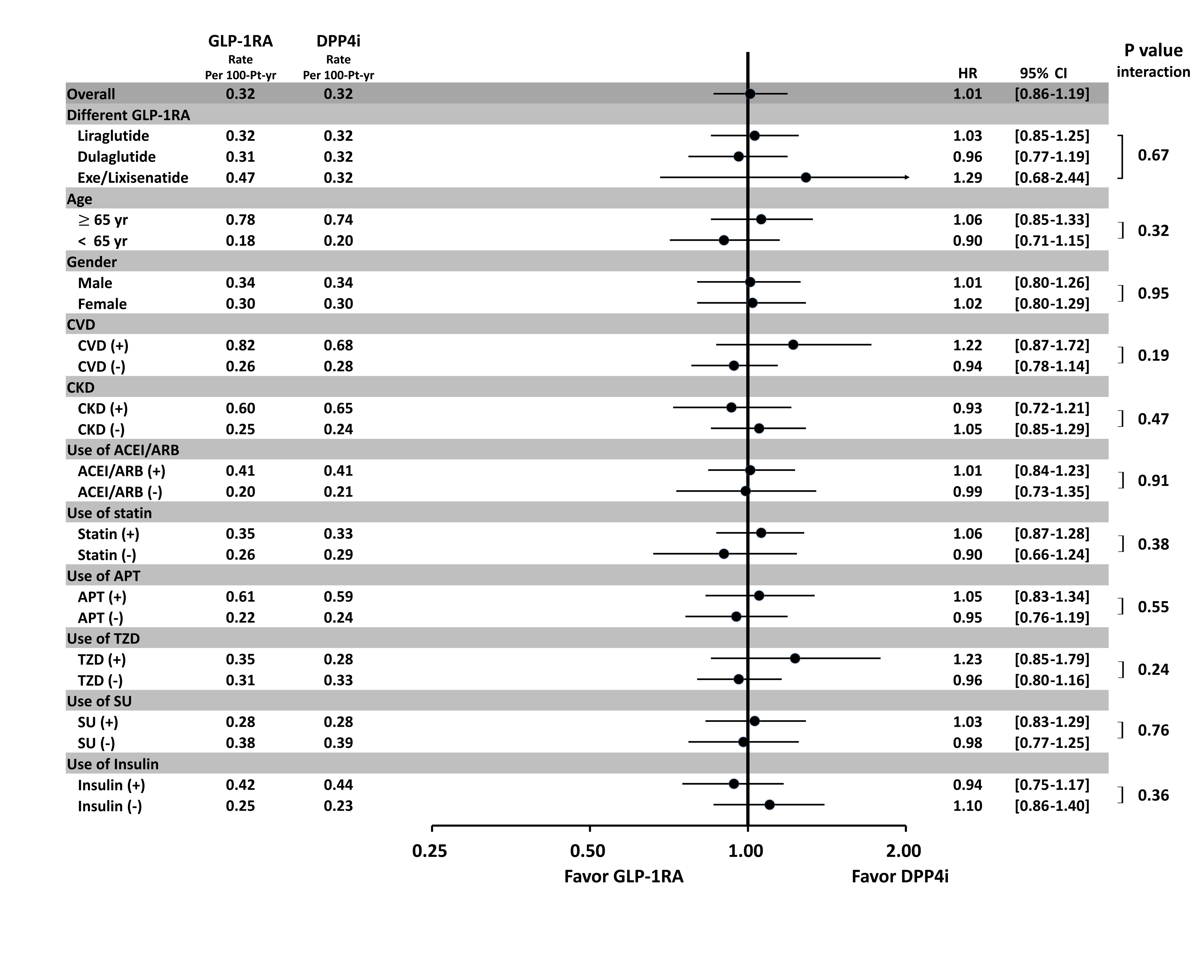

Supplement: Supplementary file 4 — Additional file 4: Figure S3. Subgroup analysis of forest plot of HR for GLP-1RA versus DPP4i among patients with T2D after PSM. There was no difference of the risk of incident AF between the GLP-1RA and DPP4i across all subgroups (P interaction > 0.05). [file 12933_2022_1549_MOESM4_ESM.tif]
